# Characteristic alterations of gut microbiota and serum metabolites in patients with chronic tinnitus: a multi-omics analysis

**DOI:** 10.1128/spectrum.01878-24

**Published:** 2024-11-18

**Authors:** Jiang Wang, Jia-Hui Xiang, Xu-Yuan Peng, Min Liu, Le-Jia Sun, Min Zhang, Li-Yuan Zhang, Zhi-Bin Chen, Zheng-Quan Tang, Lei Cheng

**Affiliations:** 1 Department of Otorhinolaryngology & Hearing International Jiangsu Ear and Hearing Center, The First Affiliated Hospital, Nanjing Medical University, Nanjing, China; 2 Department of Breast Surgical Oncology, National Cancer Center & National Clinical Research Center for Cancer, Cancer Hospital, Chinese Academy of Medical Sciences and Peking Union Medical College, Beijing, China; 3 Department of General Surgery, The First Affiliated Hospital, Nanjing Medical University, Nanjing, China; 4 School of Life Sciences, Anhui University, Hefei, China; 5 Key Laboratory of Human Microenvironment and Precision Medicine of Anhui Higher Education Institutes, Anhui University, Hefei, China; Children's National Hospital, George Washington University, Washington, DC, USA

**Keywords:** chronic tinnitus, gut microbiota, serum metabolites, gut-brain-ear axis, multi-omics analysis

## Abstract

Chronic tinnitus is a central nervous system disorder. Currently, the effects of gut microbiota on tinnitus remain unexplored. To explore the connection between gut microbiota and tinnitus, we conducted 16S rRNA sequencing of fecal microbiota and serum metabolomic analysis in a cohort of 70 patients with tinnitus and 30 healthy volunteers. We used the weighted gene co-expression network method to analyze the relationship between the gut microbiota and the serum metabolites. The random forest technique was utilized to select metabolites and gut taxa to construct predictive models. A pronounced gut dysbiosis in the tinnitus group, characterized by reduced bacterial diversity, an increased Firmicutes/Bacteroidetes ratio, and some opportunistic bacteria including *Aeromonas* and *Acinetobacter* were enriched. In contrast, some beneficial gut probiotics decreased, including Lactobacillales and Lactobacillaceae. In serum metabolomic analysis, serum metabolic disturbances in tinnitus patients and these differential metabolites were enriched in pathways of neuroinflammation, neurotransmitter activity, and synaptic function. The predictive models exhibited great diagnostic performance, achieving 0.94 (95% CI: 0.85–0.98) and 0.96 (95% CI: 0.86–0.99) in the test set. Our study suggests that changes in gut microbiota could potentially influence the occurrence and chronicity of tinnitus, and exert regulatory effects through changes in serum metabolites. Overall, this research provides new perceptions into the potential role of gut microbiota and serum metabolite in the pathogenesis of tinnitus, and proposes the “gut-brain-ear” concept as a pathomechanism underlying tinnitus, with significant clinical diagnostic implications and therapeutic potential.

**IMPORTANCE:**

Tinnitus affects millions of people worldwide. Severe cases may lead to sleep disorders, anxiety, and depression, subsequently impacting patients’ lives and increasing societal healthcare expenditures. However, tinnitus mechanisms are poorly understood, and effective therapeutic interventions are currently lacking. We discovered the gut microbiota and serum metabolomics changes in patients with tinnitus, and provided the potential pathological mechanisms of dysregulated gut flora in chronic tinnitus. We proposed the innovative concept of the “gut-brain-ear axis,” which underscores the exploration of gut microbiota impact on susceptibility to chronic tinnitus through serum metabolic profile modulation. We also reveal novel biomarkers associated with chronic tinnitus, offering a new conceptual framework for further investigations into the susceptibility of patients, potential treatment targets for tinnitus, and assessing patient prognosis. Subsequently, gut microbiota and serum metabolites can be used as molecular markers to assess the susceptibility and prognosis of tinnitus.Furthermore, fecal transplantation may be used to treat tinnitus.

## INTRODUCTION

Tinnitus, a pervasive auditory disorder affecting hundreds of millions worldwide, is characterized by the perception of sound in the absence of an external acoustic stimulus (1, 2). A global population-based meta-analysis reported a pooled prevalence of any tinnitus among adults of 14.4% (95% CI: 12.6%–16.5%) and concluded that severe tinnitus was perceived as a major problem affecting normal life by >120 million people (3). Numerous studies have indicated that chronic tinnitus significantly diminishes patients’ quality of life and is closely associated with psychosomatic symptoms such as sleep disturbances, depression, anxiety, and personality traits (4–6). However, to date, there is no effective treatment for tinnitus because of the limited understanding of its underlying mechanism (7). Although several hypotheses for tinnitus have been proposed, there is no direct evidence to confirm these theories.

Cumulative evidence indicates that the central nervous system (CNS), including all levels of auditory conduction and non-auditory pathways, such as the limbic system, plays a significant role in the development and persistence of tinnitus (8–11). The central gain control theory suggests that a reduction in afferent signal, caused by cochlear dysfunction, leads to the remodeling of auditory cortex neurons and an increase in spontaneous or synchronous firing. Eventually, subsequent abnormal excitation in the auditory cortex causes tinnitus symptoms (10–14). Other studies have also suggested that neuroinflammation plays a role in tinnitus development (15–17). Tinnitus animals show similar inflammatory characteristics, mainly including the change of expression of pro-inflammatory factors and the activation of microglia. Additionally, inhibiting or genetically mutating tumor necrosis factor alpha (TNF-α) has been shown to alleviate behavioral symptoms associated with tinnitus in animal models (18). Therefore, leading scholars believe that CNS change is the main cause of chronic tinnitus, but the specific regulation mechanism is unclear.

Currently, the gut and its microbes are one of the main factors affecting the CNS function. The term “gut-brain axis” underscores the intricate interplay between gut microbiota and the brain, mediated by the vague nerve and through endocrine and immune signaling pathways (19–21). The gut microbiota functions as an essential endocrine organ, metabolizing dietary substances and generating biologically active compounds. These compounds, including neurotransmitters and inflammation mediators, enter the peripheral circulation and influence host metabolism (22–26). Nowadays, several studies prove that gut microbiota is closely linked to disease pathogenesis, such as bipolar depression, sleeping disorders, and schizophrenia (27–29). Compared to healthy controls, patients with bipolar depression exhibit a distinct profile of abundant microbial-derived neuroactive metabolites, including gamma-aminobutyric acid and short-chain fatty acids (28). The above suggests that the gut microbiota may regulate CNS function by affecting serum metabolite changes.

Given the CNS’s involvement in tinnitus and its known interactions with gut microbiota, it is plausible to explore the gut-brain axis as a contributor to tinnitus pathogenesis. Although there is currently no study investigating the potential impact of gut microbiota on tinnitus, it has been demonstrated that dysregulation of the gut microbiota could influence the serum metabolite, production of neurotransmitters, and finally induce brain neuroinflammatory responses in CNS’s diseases (18, 30–32). This highlights the urgency of a precise mechanism linking gut microbial dysbiosis to metabolic disturbances in tinnitus (33–35). Recent advancements in multi-omics technologies have allowed for a comprehensive understanding of human diseases at the systemic level (36–41). This study aimed to characterize gut microbiota and serum metabolite profiles in patients with tinnitus, identify key microbial taxa and metabolites associated with tinnitus, and develop a predictive model for tinnitus diagnosis. We hypothesize that dysregulation of gut microbiota contributes to the pathogenesis of chronic tinnitus by modulating serum metabolites, thereby influencing neuroinflammatory pathways.

## MATERIALS AND METHODS

### Study population

The inclusion criteria for the tinnitus group were as follows: (i) subjective persistent tinnitus; (ii) duration of disease over 6 months; (iii) tinnitus frequency matching in the range of 250–8,000 Hz; (iv) participants were able to complete the scale independently and cooperate with the doctor. To avoid the disturbance of pre-exsiting factors in our analysis, we excluded patients with (i) tinnitus caused by ear disease, surgery, or trauma; (ii) disease or treatment that may affect gut microbes in the previous 6 months; (iii) use of systemic antibiotics, corticosteroids, or any other immunosuppressive therapy or oral probiotics in the previous 6 months. Each patient underwent tinnitus matching, pure-tone audiometry, the Tinnitus Handicap Inventory (THI), Self-rating Anxiety Scale (SAS), and the Pittsburgh Sleep Quality Index (PSQI). Meanwhile, this study included a healthy control (HC) group aged 18–65 years without tinnitus, with no history of disease or treatment in the previous 6 months that may affect gut microbes.

### Fecal sample collection and 16S rDNA sequencing

Five grams plus minus 0.1 g of fresh stool samples were collected from each patient and stored at −80°C. DNA was extracted using cetyltrimethylammonium bromide, which was subsequently stored at −80°C until PCR measurement was performed. Ultrapure water was used as the negative control during DNA extraction to prevent false-positive PCR results.

Primers were tagged with barcodes and universal primers. PCR was performed using 25 ng template DNA, with primers based on the V3-V4 segment (341F and 805R). The amplification conditions included (i) denaturation at 98°C for 30 seconds; (ii) 32 cycles of denaturation (98°C, 10 seconds), annealing (54°C, 30 seconds), and extension (72°C, 45 seconds); (iii) final extension at 72°C for 10 min. The PCR products, confirmed by agarose gel electrophoresis, were purified using AMPure XP beads (Beckman Coulter Genomics, Danvers, MA, USA) and quantified with Qubit (Invitrogen, USA) before preparing amplicon pools for sequencing. The sizes and quantities of the PCR products were assessed using an Agilent 2100 Bioanalyzer (Agilent, USA) and a Library Quantification Kit for Illumina (Kapa Biosciences, Woburn, MA, USA), respectively. Finally, libraries were sequenced using the NovaSeq PE250 platform (LC-Bio Technologies Co., Ltd., China).

### Microbiome data analysis

Samples were sequenced on an Illumina NovaSeq platform (LC-Bio Technologies Co., Ltd., China). The sequence quality was great as shown in Table S1 and the Q30 of all samples was >90%. Paired-end reads were assigned to samples based on their unique barcodes, merged using FLASH (v.1.2.8, parameter: “-m 10 -M 100 -x 0.25 -t 1 -z,” USA), and quality-filtered to obtain high-quality clean tags. Chimeric sequences were filtered using the Vsearch software (v.2.3.4, China). A feature table and sequences were obtained after dereplication with DADA2 (v.1.14). Alpha and beta diversities were calculated using QIIME2 (Qiime2. 2022.8) and normalized to the same sequences. The feature abundance was normalized using the SILVA classifier (Release 138, https://www.arb-silva.de/documentation/release-138/; annotation threshold: --min_confidence 0.7). Blast was used for sequence alignment and annotation with SILVA database (https://www.arb-silva.de/). PICRUSt2 (https://github.com/picrust/picrust2) predicted metagenomic functional composition. Further diagrams were created using R (v.3.5.2).

### Blood sampling and plasma metabolomics analysis

Peripheral blood samples were collected from all participants in anticoagulation tubes and were temporarily stored at 4°C for less than 30 min. Furthermore, they were centrifuged for 15 min at 2,000 rpm and plasma was stored at −80°C until further detection. Liquid chromatography-mass spectrometry (LC-MS, Thermo Fisher Scientific, USA) was performed to analyze plasma metabolites. The metabolites were extracted with 80% precooled methanol buffer and the extraction mixtures were stored for 30 min at −20°C. After centrifugation at 20,000 *g* for 15 min, the supernatants were transferred to new tubes and vacuum-dried. These samples were redissolved with 100 µL 80% methanol and stored at −80°C before the LC-MS analysis. In addition, pooled quality control (QC) samples were also prepared by combining 10 µL of each extraction mixture. All samples were analyzed using an LC-MS system, according to the manufacturer’s instructions.

The MS data were processed using XCMS software (v.3.4.1 [42]) to pick peaks, group them, correct retention times, and annotate isotopes and adducts. Raw LC-MS data files were converted to mzXML format and then analyzed with R software using XCMS, CAMERA (v.3.4.1 [43]), and the metaX toolbox (v.1.4.16 [44]). Each ion was identified by combining retention time and m/z data. The intensity of each peak was recorded in a three-dimensional matrix containing arbitrarily assigned peak indices (retention time-m/z pairs), sample names (observations), and ion intensity information (variables).

The online Kyoto Encyclopedia of Genes and Genomes (KEGG, v.98.0) and Human Metabolome Database (HMDB, v.4.0) were used to match the molecular mass data of samples with those in the database. If the mass difference was less than 10 ppm, the metabolite was annotated and its molecular formula was further identified through isotopic distribution measurements. We also used an in-house fragment spectrum library to validate the metabolite identification.

The raw protein intensity was normalized using the “medium” method. Hierarchical clustering and principal component analysis (PCA) were performed using the Pheatmap and metaX packages. Partial least squares discriminant analysis (PLS-DA) was conducted using the R package ropls to calculate the variable importance in projection (VIP) values for each variable. Correlation analysis was performed using Pearson’s correlation coefficient using the cor package. The final metabolites with significant differences were screened based on three conditions: *P*-value <0.05, difference multiple >1.2 or <0.83 obtained by *t*-test, and VIP >1 calculated via PLS-DA.

Hypergeometric-based enrichment analysis with KEGG pathway was used to annotate protein sequences. Gene set enrichment analysis (GSEA) and the Molecular Signatures Database were used to compare gene sets in different situations. A significant difference between the two groups was determined by |normalized enrichment score (NES)| > 1 and nominal (NOM) *P*-values of <0.05. A network map was drawn based on the metabolic pathways.

### Microbiome-metabolome correlation analysis

To ensure a one-to-one correspondence between metabolites and gut microbes, we analyzed the omics data of subjects who underwent both metabolomic and microbiome assays for combined analyses. Weighted gene co-expression network analysis (WGCNA) was utilized to consolidate metabolites and microbiota into modules, utilizing a newly developed computational platform (45). Cluster correlations were analyzed using OmicStudio (https://www.omicstudio.cn) The Mantel test was performed to identify microbiota-related metabolites.

### Statistical analyses

Statistical analyses were performed using R (version 4.0.0). Except for the prediction of the relative abundance of aerobic bacteria based on the BugBase database [Mann-Whitney-Wilcoxon test with false discovery rate (FDR) corrected, *P* < 0.01], the statistical significance of other analyses was set at *P* < 0.05. The demographic characteristics of the participants and the abundance of pathways that differed between the tinnitus and HC groups were analyzed using *t*-tests. In microbiome analysis, due to the diverse and intricate nature of the samples, microbiome data often deviate from a normal distribution, thus necessitating non-parametric testing methods. Alpha-diversity was analyzed using the Wilcoxon rank-sum test. Significant difference test was analyzed using the Mann-Whitney U-test. PICRUSt2 analysis based on KEGG pathway annotation was analyzed based on Stamp differential analysis (*t*-test). Linear discriminant analysis effect size (LEfSe) was analyzed using the Kruskal-Wallis rank-sum test and the Wilcoxon rank-sum test. *T*-test was used in all metabolic analyses. To standardize the data, the raw metabolite abundances were log-transformed prior to analysis, and it is generally believed that the standardized data follow a normal distribution. In the analysis between the microbiota (metabolite) and clinical traits, Spearman’s correlation analysis was used since the clinical characteristics of patients are classified as variables.

Further differential microbiome and metabolome modules clustered by WGCNA were identified between tinnitus and control groups using the Wilcoxon rank-sum test with the Benjamini-Hochberg method to control for FDR. Also, FDR correction was used for the Pearson’s test when analyzing the correlation between those tinnitus-related microbiome and metabolite modules. The random forest (RF) technique, as a classifier that trains and predicts the samples using multiple trees, was utilized to determine the number of incorporated variables, with variable importance indicated by the mean decrease in the Gini index. The performance of the RF model was evaluated by calculating the area under curve (AUC) of the receiver operating characteristic (ROC) curve. Furthermore, 10,000 bootstrap samples were used in the test set to generate a 95% CI for area under the receiver operating characteristic curve (AUROC) in the test set. The entire test set was used to calculate the AUROC point estimate.

## RESULTS

### Study population characteristics

Seventy patients with tinnitus and 30 healthy volunteers were included in this study. The demographic and clinical characteristics of the participants are summarized in Table 1. The average age and percentage of female participants in the tinnitus group were higher than those in the HC group; however, the differences were insignificant (*P*-value >0.05). There was no difference in body mass index (BMI) between the two groups. In the tinnitus group, the average duration of tinnitus was 6.84 years, with most patients (74.29%) experiencing bilateral tinnitus. Most patients exhibited moderate loudness and high-frequency symptoms following tone-matching tests for dominant tinnitus. Eight patients reported disabling levels of severity according to the THI scale (five with severe disability and three with catastrophic disability). Results from the SAS and PSQI indicated that most patients with tinnitus experienced mild to moderate anxiety and good sleep quality, with only three subjects reporting very poor sleep quality.

**TABLE 1:** Demographic and clinical characteristics of participants^*a*^

Baseline features	Tinnitus patients (*n* = 70)	Healthy group (*n* = 30)	*P*-value
Age (years), mean ± SD	44.61 ± 12.26	40.50 ± 11.55	0.12
Sex: male, *n* (%)	39 (55.71)	13 (43.3)	0.19
BMI (kg/m^2^), mean ± SD	23.98 ± 3.64	23.11 ± 3.16	0.26
Time since diagnosis of tinnitus (years), mean ± SD	6.84 ± 6.20	–^*b*^	
Side of tinnitus: bilateral, *n* (%)	52 (74.29)	–	
Tinnitus loudness (dB HL), mean ± SD	41.04 ± 15.69	–	
Grade I (≤25 dB HL), *n* (%)	20 (28.57)	–	
Grade II (26–40 dB HL), *n* (%)	17 (24.29)	–	
Grade III (41–55 dB HL), *n* (%)	23 (32.86)	–	
Grade IV (56–70 dB HL), *n* (%)	8 (11.43)	–	
Grade V (≥71 dB HL), *n* (%)	2 (2.85)	–	
Tinnitus frequency (kHZ), median (IQR)	8,000 (4,000–8,000)	–	
Grade I (≤0.75 kHZ), *n* (%)	5 (7.14)	–	
Grade II (1.0–3.0 kHZ), *n* (%)	4 (5.71)	–	
Grade III (≥4.0 kHZ), *n* (%)	61 (87.15)	–	
Score of THI, mean ± SD	35.26 ± 19.09	–	
Grade I (≤16), *n* (%)	9 (12.86)	–	
Grade II (18–36), *n* (%)	33 (47.14)	–	
Grade III (38–56), *n* (%)	20 (28.57)	–	
Grade IV (58–76), *n* (%)	5 (7.14)	–	
Grade V (78–100), *n* (%)	3 (4.29)	–	
Score of SAS, mean ± SD	40.21 ± 8.19	–	
Grade I (≤49), *n* (%)	62 (88.58)	–	
Grade II (50–59), *n* (%)	5 (7.14)	–	
Grade III (60-69), *n* (%)	3 (4.28)	–	
Grade IV (≥70), *n* (%)	0 (0)	–	
Score of PSQI, mean ± SD	6.33 ± 4.13	–	
Grade I (≤5), *n* (%)	37 (52.86)	–	
Grade II (6–10), *n* (%)	23 (32.86)	–	
Grade III (11–15), *n* (%)	7 (10.00)	–	
Grade IV (≥16), *n* (%)	3(4.22)	–	

^*a*^ HL, hearing level. IQR, interquartile range.

^*b*^ –, no data.

### Gut microbiome analysis

**Fig 1:**
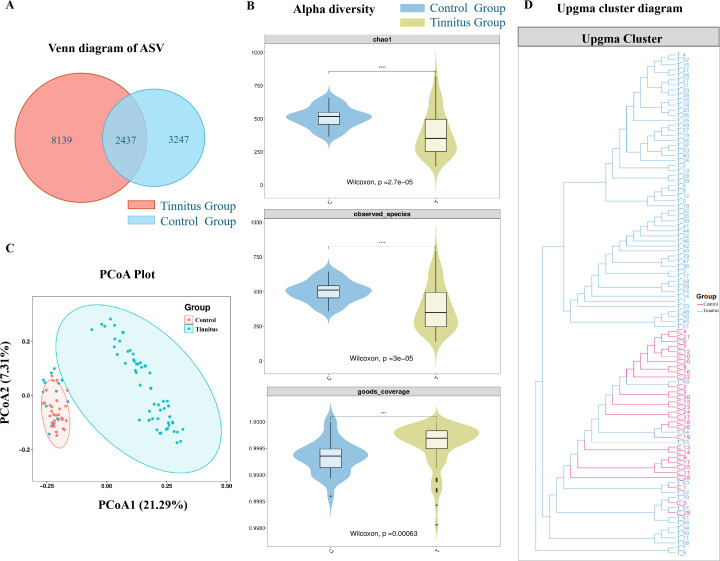
Gut microbiome dysbiosis in tinnitus group. (A) A Venn diagram demonstrating the existence of ASVs in the tinnitus and healthy groups. The area where two circles intersect indicates the number of ASVs shared between the two groups. (B) Significant differences existed in alpha-diversity between the two groups, including Chao1, observed_otus, and goods_coverage. Wilcoxon rank-sum test was used. (C) The PCoA was performed using an unweighted UniFrac matrix. (D) UPGMA cluster diagram. The UPGMA is based on unweighted UniFrac metrics.

In total, 13,823 amplicon sequence variants (ASVs) were identified. Figure 1A shows the presence of 8,139 and 3,247 group-specific ASVs in the tinnitus and HC groups, respectively. Alpha-diversity analysis (Fig. 1B) revealed a reduction in species richness (Chao1) in the gut microbiota of patients with tinnitus compared with that in the HC group. Furthermore, principal coordinates analysis (PCoA) with unweighted UniFrac metrics was used to calculate the similarity between samples and transform high-dimensional data into low-dimensional representations (Fig. 1C). PCoA showed a significant difference in the gut microbiota composition between patients with tinnitus and the HC group (*R* = 0.291, *P* = 0.001). Additionally, the Unweighted Pair Group Method with Arithmetic Mean (UPGMA) based on unweighted UniFrac metrics also indicated substantial discrimination between the groups, suggesting an altered overall microbial composition in patients with tinnitus (Fig. 1D).

**Fig 2:**
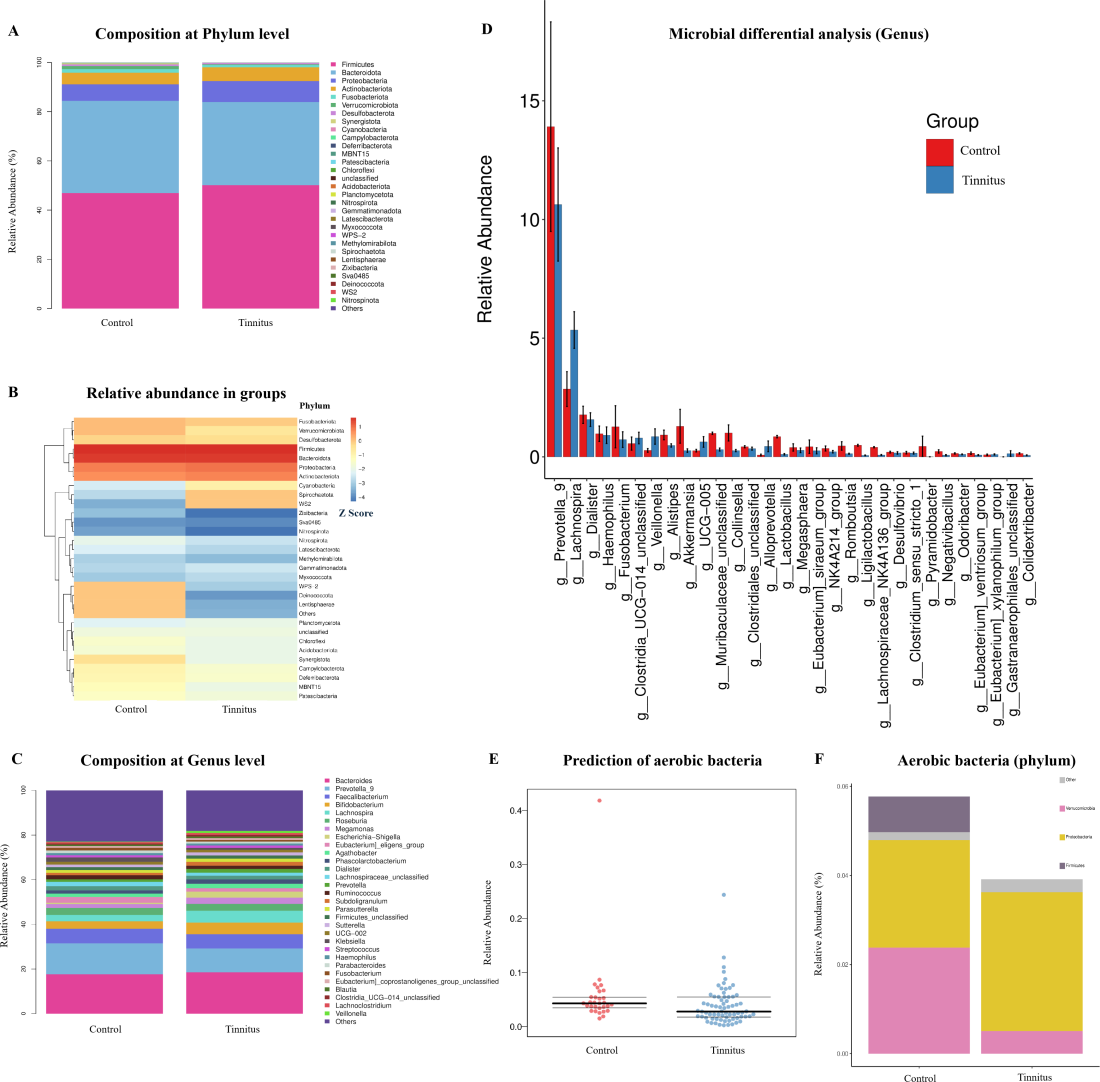
Overview of the gut microbiome in tinnitus and HC groups. (**A**) Composition of these two groups at the phylum level. Dominant phyla and their relative abundance in each group were exhibited. (**B**) Relative abundance in tinnitus and control groups. Each row represents a bacterium (top 30), and each column represents groups. At the phylum level, the expression abundance of the same bacterium is normalized by Z-score. It is possible to compare the same microorganism between different groupings. (**C**) Composition of these two groups at the genus level. Dominant genus and their relative abundance in each group. (**D**) Genus-level microbial differential analysis. Mann-Whitney U-test. A bar chart displaying the top 30 bacterium with the highest relative abundance with *P* < 0.05. The horizontal axis represents the differential species (arranged from left to right in order of abundance), and the vertical axis represents relative abundance. (**E**) Relative abundance of aerobic bacteria predicted based on the BugBase database. *P* < 0.01. Mann-Whitney-Wilcoxon test with FDR corrected. (**F**) Distribution of aerobic bacteria at the phylum level in each group.

The relative abundances of the gut microbiome at the phylum and genus level are shown in Fig. 2A and C, respectively. More than 90% of fecal bacteria belonged to phyla Firmicutes (50.06% vs 46.92%), Bacteroidetes (33.88% vs 37.49%), Proteobacteria (8.44% vs 6.71%), and Actinobacteria (5.66% vs 4.71%) in tinnitus and control groups, respectively. The top three dominant genera identified in both the tinnitus and HC group were consistent, including Bacteroides (18.50% vs 17.60%), *Prevotella_9* (10.63% vs 17.60%), and *Faecalibacterium* (6.45% vs 6 .45%). As shown in Fig. 2B, several gut taxa exhibited different abundance patterns in these two groups at the phylum level. Significantly different gut taxa at the genus level between tinnitus and healthy groups were shown in Fig. 2D. The relative abundances of the genera *Prevotella*, *Fusobacterium*, *Alistipes*, and *Akkermansia* decreased in the tinnitus group. In contrast, the relative abundance of *Lachnospira* was significantly higher than that in the HC group. Based on predictions from the BugBase database, we assessed the phenotypes of the gut flora in these two groups. As depicted in Fig. 2E, there was a significant decrease in the sum of aerobic bacteria in the tinnitus group compared with that in the HC group. The relative abundances and distributions of anaerobic bacteria at the phylum level in both groups are shown in Fig. 2F.

Furthermore, Wilcoxon rank-sum tests were conducted to identify the different gut taxa in the tinnitus group, with a threshold of q-value <0.05 and |log2FC| > 2. Notably, 70 significantly differential taxa at the genus level were shown in Table S2, with 18 genera enriched in patients with tinnitus and 52 depleted. *Aeromonas* and *Acinetobacter*, both opportunistic pathogens (46, 47), were notably more abundant in the tinnitus group. *Rhodococcus* were significantly more abundant in the tinnitus group, whereas a significant depletion of *Akkermansia* was observed. Furthermore, *Lactobacillus* and *Lactococcus*, known probiotics that play a role in maintaining host immunity homeostasis (48, 49), were significantly enriched in the HC group.

Using Spearman’s correlation analysis with a threshold of *P*-value <0.05, we determined the correlation between the genera and clinical phenotypes. Notably, *Weissella*, a member of the lactic acid bacteria group with demonstrated probiotic and anti-inflammatory potential in recent studies (50), exhibited a negative association with the THI score, indicating an inverse relationship with tinnitus severity. In contrast, *Rhodococcus* and *Phyllobacterium*, which were enriched in individuals with tinnitus, were positively correlated with anxiety levels and sleep disturbance.

**Fig 3:**
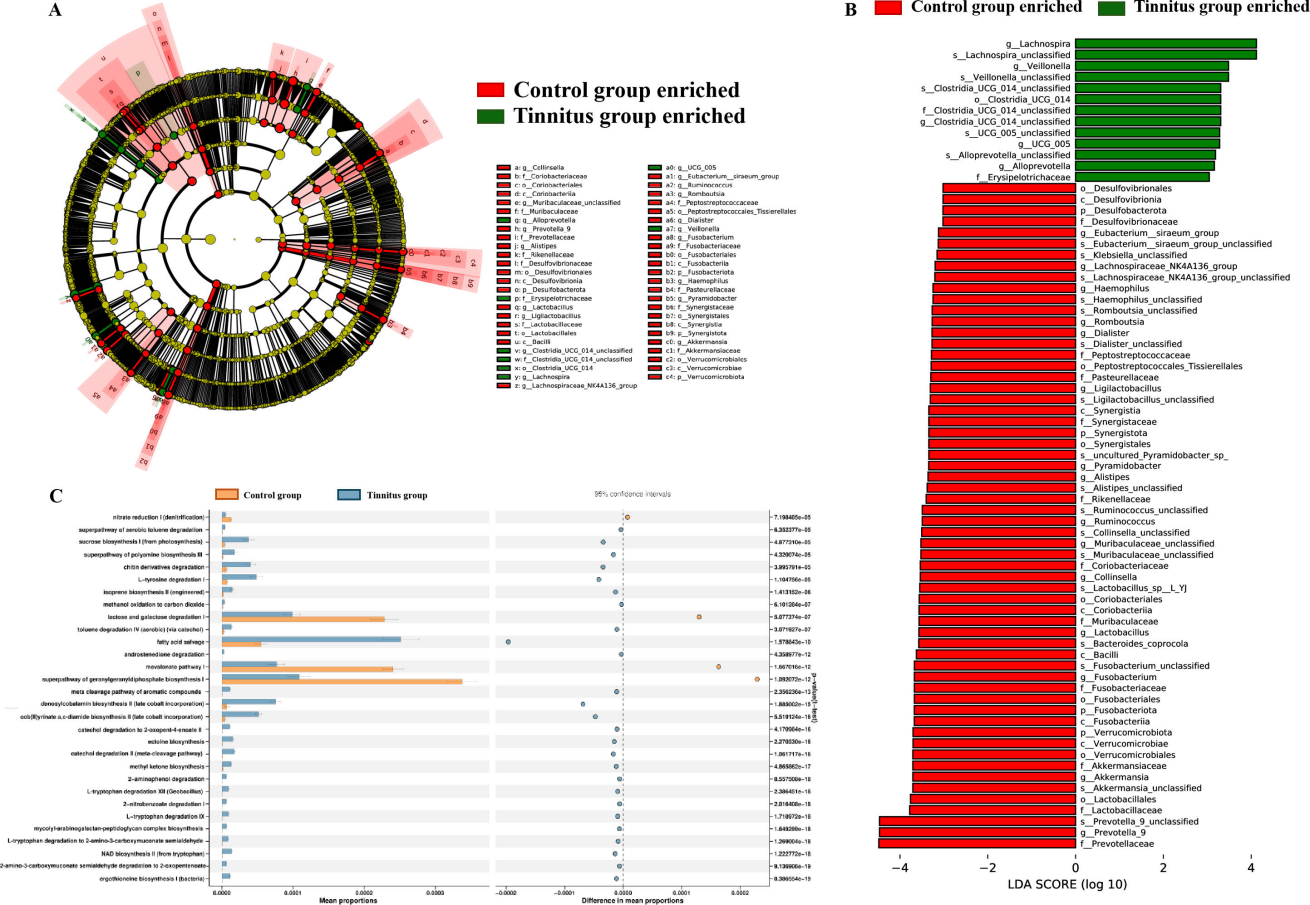
Biomarker of gut microbiota and their predicted function. (**A**) Cladogram of gut microbiota. The concentric circles radiating outward represent the seven taxonomic levels of genus, species, order, family, class genus, order genus, and species-genus, respectively, starting from the innermost circle. Each node represents a species classification at this level, with the species abundance represented by the node’s size. Each dot represents a taxonomic hierarchy and is marked for significant (LEfSe: *P* < 0.05) enrichment either in the tinnitus group (green) or in the HC group (red). (**B**) Histogram of distribution. Taxa that reached a LDA score >3.0 are listed. The colors in the bar chart indicate the relative abundance of distinct species across different groups. In contrast, the length of the bars reflects the LDA score, which quantifies the degree of significant differences among species between groups. (**C**) PICRUSt2 analysis based on KEGG pathway annotation. *P* < 0.05. The two groups calculated *P*-value using Stamp differential analysis (*t*-test).

An LEfSe was conducted to screen for microbiota with significant differences in abundance between the two groups to identify the appropriate biomarkers. As depicted in Fig. 3A, the microbial species labeled red were enriched in the HC group, whereas those labeled green were enriched in the tinnitus group. Potential biomarkers with linear discriminant analysis (LDA) values >3 are shown in Fig. 3B. Considering the potential function of gut bacteria, we utilized PICRUSt2 to predict the representative pathways related with gut taxa, and the top 30 most significant pathways were shown in Fig. 3C. Notably, several pathways such as fatty acid salvage and L-tryptophan degradation were significantly upregulated in the tinnitus group. Conversely, pathways were significantly downregulated, including lactose and galactose degradation I and mevalonate pathway I.

### Differential serum metabolites

**Fig 4:**
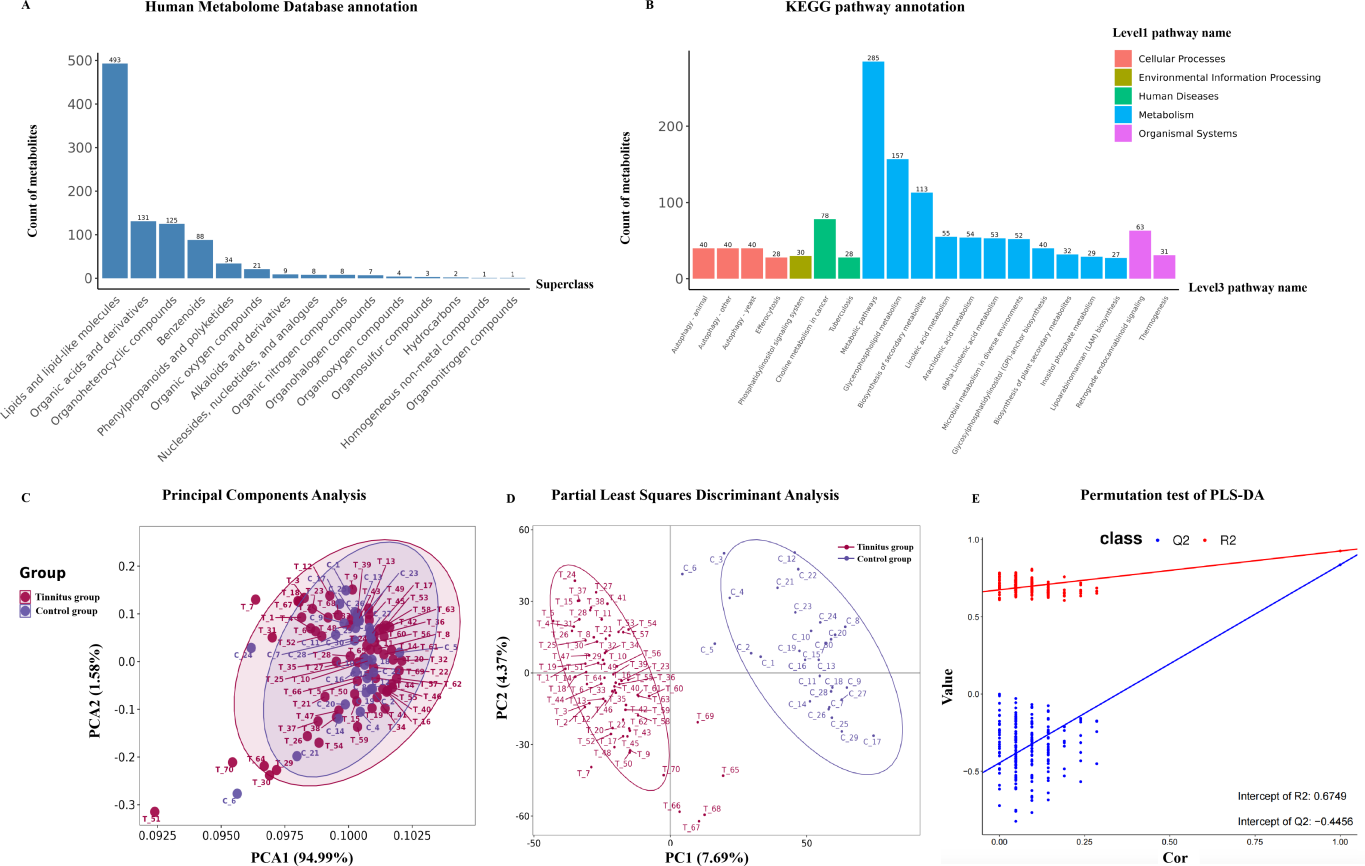
Dysregulation of serum metabolic profile in tinnitus group. (**A**) Histogram reveals the counts of metabolites annotated by HMDB at the superclass level. The horizontal axis represents the superclass classification of metabolites. The vertical axis represents the number of metabolites contained in that classification. (**B**) Histogram reveals the counts of metabolites annotated by the KEGG pathway at level 3. The horizontal axis represents the level 3 classification. The vertical axis represents the number of metabolites in that classification. Different colors indicate different level 1 classifications. (**C**) The PCA performed to reduce dimension and discriminate serum metabolic profile between tinnitus (red) and control (purple) groups. The horizontal axis represents the first principal component. The vertical axis represents the second principal component. The numbers in parentheses indicate the degree to which each principal component explains the overall data. (**D**) The PLS-DA model discriminates individuals. Tinnitus patients were labeled in red. Healthy individuals were labeled in purple. The horizontal axis represents the first principal component. The vertical axis represents the second principal component. The numbers in parentheses indicate the degree to which each principal component explains the discriminative model. (**E**) Permutation test indicated that the model has not overfit. The R2 regression line was labeled in red and Q2 line was labeled in blue. The intercept of the Q2 regression line with the y-axis was shown in the bottle.

Plasma samples were analyzed using liquid LC-MS. Pearson correlation analysis performed on QC samples revealed a correlation coefficient of *R* > 0.9 , indicating the high reproducibility of the experiments. A total of 1,059 metabolites were identified [471 in positive ion mode (POS) and 588 in negative ion mode (NEG)], of which 748 were classified in the HMDB and 524 in the KEGG. The counts of metabolites annotated by HMDB at the superclass level are presented in Fig. 4A, with lipids and lipid-like molecules accounting for the majority at *n* = 493 (65.9%), followed by organic acids and derivatives at *n* = 131 (17.5%), and organoheterocyclic compounds at *n* = 125 (16.7%). The number of metabolites annotated by the KEGG pathway at level 3 is presented in Fig. 4B. The top three pathways were metabolic pathways (*n* = 285, 54.4%), glycerophospholipid metabolism (*n* = 157, 30.0%), and biosynthesis of secondary metabolites (*n* = 113, 21.6%).

The metabolites and their abundance in the different groups were presented in Fig. 4. Although PCA did not clearly distinguish between the two groups (Fig. 4C), the PLS-DA revealed significant discrimination between the tinnitus and HC groups (Fig. 4D) (R2 = 0.934, Q2 = 0.867), indicating evident metabolic disturbances under tinnitus conditions. The validation plot confirmed the robustness of the PLS-DA model (Fig. 4E). When the x-axis falls within [0,1], and the R2 regression line is positioned above Q2, with the intercept of the Q2 regression line on the y-axis being <0, it suggests that the model has not experienced overfitting.

**Fig 5:**
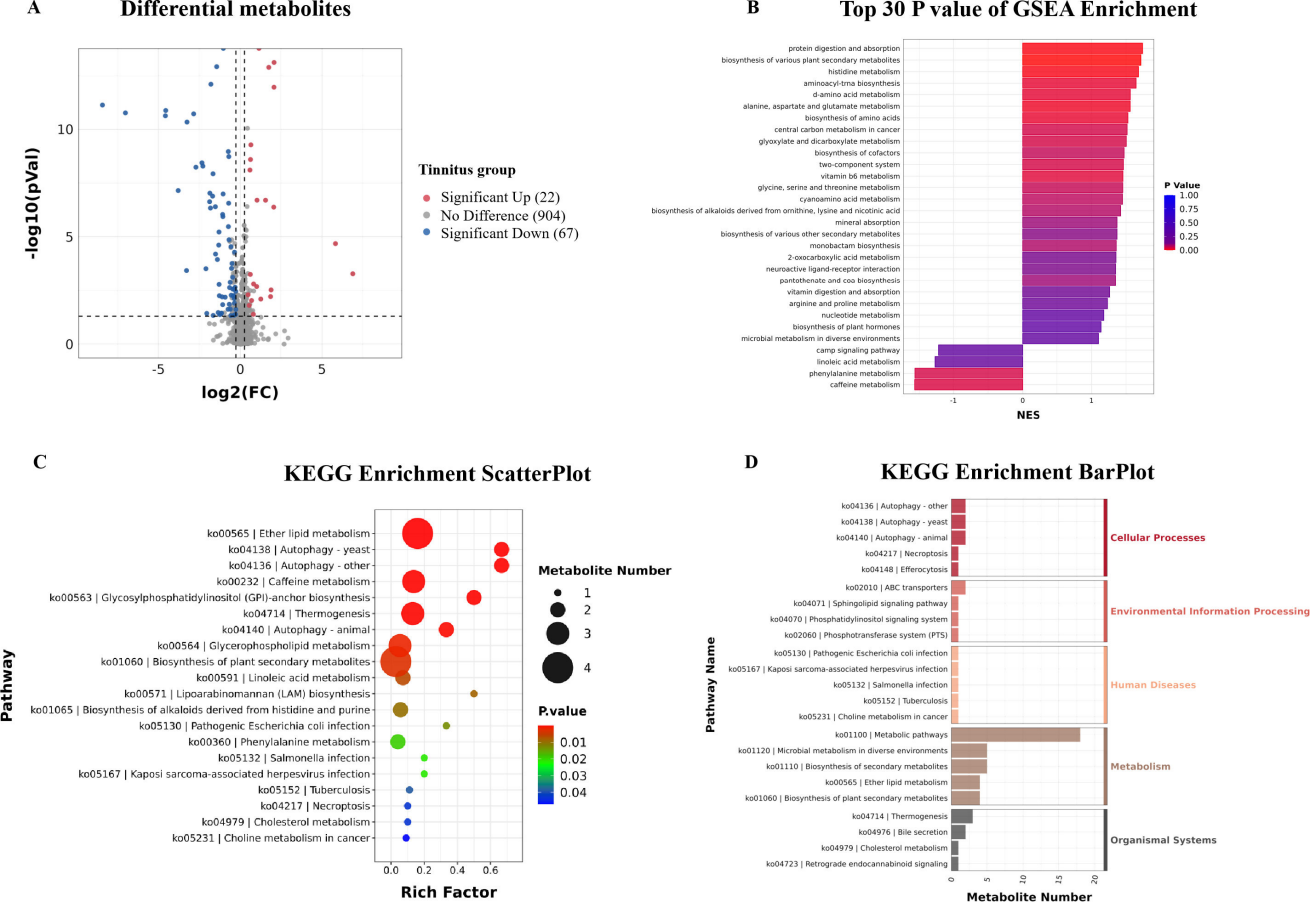
Differential serum metabolites and their enriched pathway. (**A**) Volcano plot showed the final metabolites with significant difference (*P* < 0.05, fold change >1.2 or <0.83 by *t*-test, and VIP > 1 calculated via PLS-DA) between tinnitus and control groups. The horizontal axis represents the log_2_(fold change). The vertical axis represents the -log_10_(*P*-value) obtained by *t*-test for the metabolite abundance in the two groups. Each point represents a metabolite, with red indicating a significant upregulation in the tinnitus group, blue indicating a significant downregulation, and gray indicating no significant difference. (**B**) GSEA and the Molecular Signatures Database are used for gene enrichment analysis. The top 30 significant pathways are shown in the plot, with the horizontal axis representing the NES value of the metabolite set and the vertical axis representing the pathway’s name. The color represents the NOM *P*-values. A significant difference between the two groups was determined by |NES| > 1 and NOM *P*-values of <0.05. (**C**) Scatter plot shows the KEGG pathway enriched by differential serum metabolites. The horizontal axis typically represents the enrichment score of the pathway. The vertical axis represents the name of the pathway. The size of the points represents the number of metabolites enriched, while the color indicates the *P*-value. (**D**) KEGG enrichment bar plot. The horizontal axis represents the number of metabolites enriched in the metabolic pathway. The vertical axis represents the name of the metabolic pathway. The length of each bar corresponds to the number of metabolites, and the color indicates the KEGG classification of the metabolic pathway.

We identified 89 differential metabolites (22 upregulated and 67 downregulated) in the sera of the tinnitus compared with HC groups (Fig. 5A). Spearman’s correlation analysis was conducted to examine the relationship between differential metabolites and the severity of tinnitus, as assessed by clinical methods. A total of 23 metabolites were found to correlate with indicators of disease severity (THI score, degree of anxiety, and sleep disturbance) and clinical features of tinnitus (loudness and frequency). Furthermore, six of the nine severity-related metabolites were identified as lipids and lipid-like molecules.

We focused on the functional pathways of the differentially expressed metabolites between the groups through KEGG pathway enrichment analyses. As depicted in Fig. 5C and D, ether lipid metabolism, glycerophospholipid metabolism, and linoleic acid metabolism markers exhibited significant modifications (*P* ≤ 0.01). Other metabolite sets, such as phenylalanine and cholesterol metabolism, were also enriched, although these changes were less significant (*P* > 0.01). Furthermore, GSEA (Fig. 5B) revealed significant differences in pathways related to inflammatory regulation, including tryptophan metabolism (51, 52), linoleic acid metabolism (53), biosynthesis of unsaturated fatty acids (54, 55), arachidonic acid metabolism (56), and alpha-linolenic acid metabolism (57). Other pathways associated with neuroprotective metabolites, neurotransmitter activity, and synapse function have also been implicated, such as serotonergic synapses, vitamin B6 metabolism (58), and neuroactive ligand-receptor interactions. These findings suggest substantial metabolic dysregulation in the serum of patients with tinnitus, particularly in those related to the nervous system and inflammatory balance.

### Correlation between microbiota and metabolites

**Fig 6:**
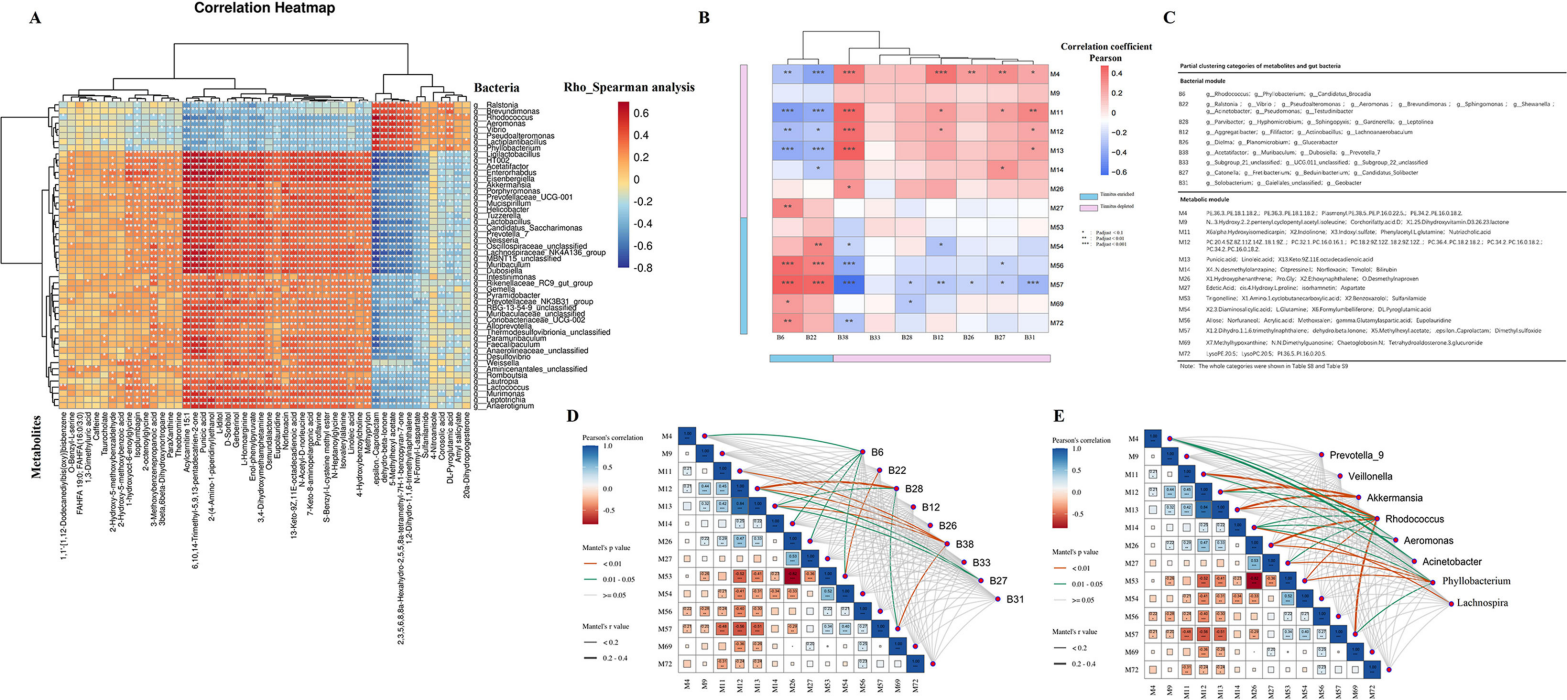
Correlation analysis results between gut bacteria and serum metabolites. (**A**) The Spearman’s correlation coefficient for differential gut microbiota and serum metabolites between the two groups. The color represents the correlation coefficient (rho) in Spearman’s analysis. “*” is labeled for *P* < 0.05. “**” is labeled for *P* < 0.005. (**B**) Correlation analysis between tinnitus-related metabolic modules and bacterial modules generated by WGCNA. The left and bottom panels show associations between clusters and tinnitus. Blue indicates that modules enrich in tinnitus group, and pink indicates depletion. The right panel shows associations between the metabolomic clusters and bacterial clusters. Pearson’s test. The heatmap color represents the correlation coefficients, and the labels for the *P*-value are shown on the right. (**C**) Provided detailed information on partial gut bacteria and serum metabolites in tinnitus-related clusters generated by WGCNA. FDR-corrected *P*-value <0.05. Wilcoxon rank-sum test. (**D and E**) Inner pairwise comparisons of tinnitus-related metabolic clusters were shown, with a color gradient denoting Pearson’s correlation coefficient. Tinnitus-related bacteria clusters and specific gut microbiota are related to each metabotypes by Mantel tests. Edge width corresponded to the Mantel’s *r* statistic for the corresponding metabotypes, and edge color denoted the statistical significance.

Spearman’s correlation analysis examined the relationship between tinnitus-associated gut microbiota and dysregulated serum metabolites. As shown in Fig. 6A, it demonstrated a strong correlation between the gut microbiota associated with tinnitus and dysregulated serum metabolites. Genera enriched in the tinnitus group and positively correlated with disease severity, such as *Rhodococcus* and *Phyllobacterium*, exhibited positive correlations with tinnitus-enriched serum metabolites but negative correlations with tinnitus-depleted serum metabolites. Conversely, genera enriched in the HC group, including *Prevotella 7*, *Lactobacillus*, *Lactococcus*, and *Akkermansia*, were significantly positively correlated with tinnitus-depleted serum metabolites and negatively correlated with tinnitus-enriched serum metabolites.

Using a newly developed computational platform, WGCNA was utilized to consolidate metabolites and microbiota into modules. The Pearson’s test with FDR correction was conducted to identify tinnitus-related modules, which were further analyzed for cross-domain associations (Fig. 6B). The information on individual metabolites and gut microbiota within some clusters was presented in Fig. 6C. Bacterial modules B6 and B22 were found to be enriched in patients with tinnitus and exhibited a negative correlation with M1, M12, and M13, which consist of lipid molecules and are enriched in the HC group. Interestingly, the gut microbiota within B6 and B22, including *Rhodococcus* and *Phyllobacterium*, also correlated with the clinical phenotype of tinnitus. Additionally, we identified modules M56 and M57 that showed a highly positive correlation with B6 and B22 while being enriched in individuals with tinnitus. The Mantel test was performed on bacterial modules and tinnitus-related genus to identify bacteria-related metabotypes. As shown in Fig. 6D and E, M4, M12, and M13 were highly associated with gut microbiota.

### Diagnostic performance of predictive models

**Fig 7:**
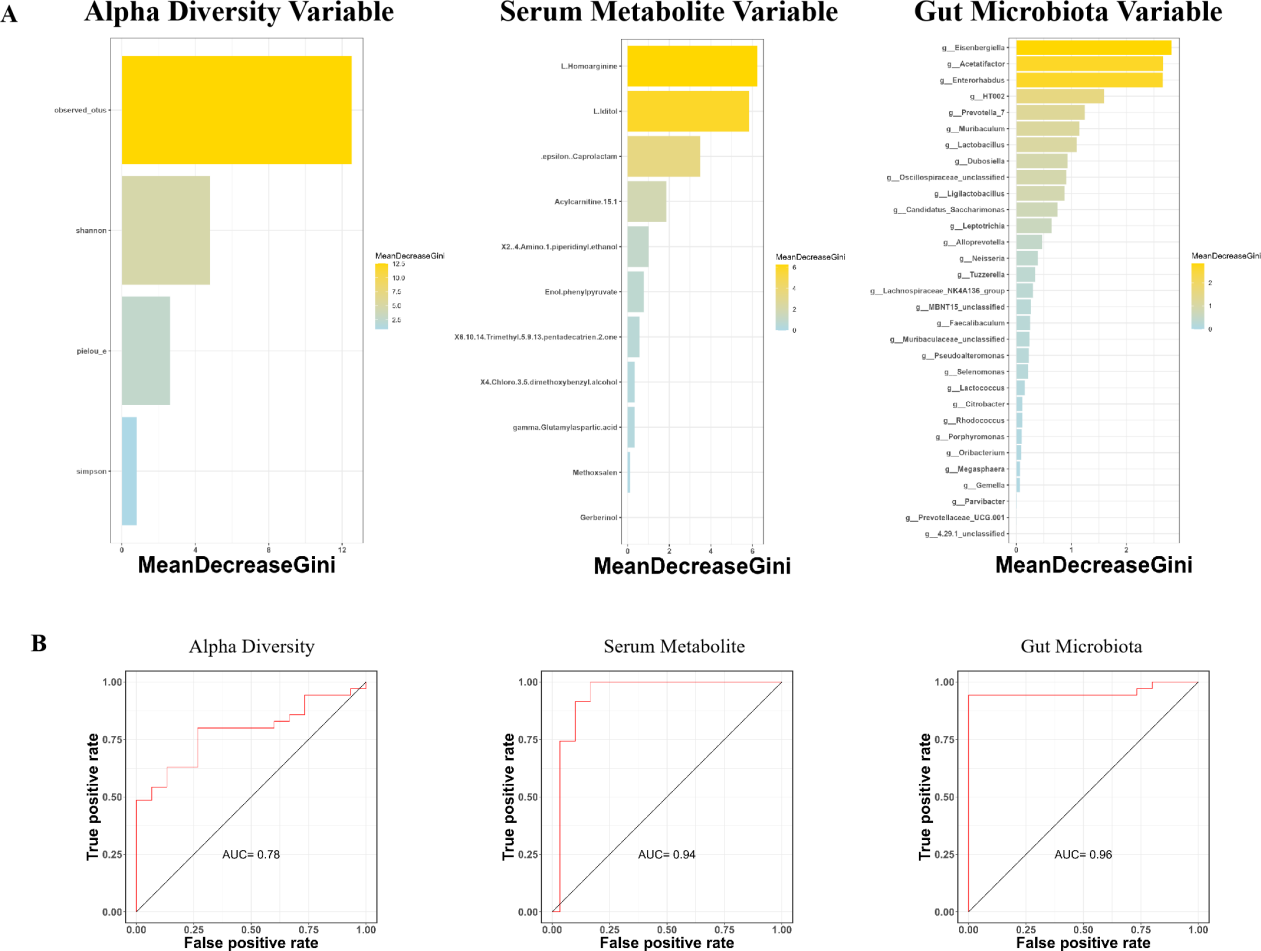
Diagnostic performance of the predictive model. (**A**) Mean decrease in Gini of the selected variables. The panels display the selected alpha-diversity index, serum metabolites, and gut microbiota from left to right. The horizontal axis represents MeanDecreaseGini, which calculates each feature’s average decrease in the Gini Index when building a decision tree. Specific features are shown in the vertical axis. (**B**) The performance of the RF models were evaluated by calculating the AUC of the ROC curve.

The RF model was utilized to identify biomarkers for diagnosing tinnitus, using selected alpha-diversity, serum metabolites, and gut taxa. The mean decrease in Gini of the selected variables is shown in Fig. 7A. The diagnostic performance of the predictive models was assessed using AUCs, with serum metabolites and gut microbiota achieving 0.94 (95% CI: 0.85–0.98) and 0.96 (95% CI: 0.86–0.99) in the test set, respectively (Fig. 7B). The alpha-diversity predictive model demonstrated limited diagnostic ability, with an AUC of 0.78 in the test set.

## DISCUSSION

In this study, we conducted an integrated multi-omics analysis of the gut microbiota, serum metabolic profiles, and clinical phenotypes of patients with chronic tinnitus. To our knowledge, this study represents the first investigation of the relationship between gut microbiota and chronic tinnitus, providing insights into the disruptions in the gut microbial composition that affect the central auditory center by modulating serum metabolic profiles. Based on our findings, we introduce the innovative concept of the “gut-brain-ear axis,” emphasizing the investigation into the gut microbiota influence susceptibility to chronic tinnitus by modulating the serum metabolic profile. The findings also identify new biomarkers associated with chronic tinnitus, providing a new conceptual framework for further research on patient susceptibility and potential treatment targets for tinnitus.

“Gut microbiota” refers to the community of trillions of commensal and symbiotic microorganisms in the human intestines (59). According to previous research, individuals with reduced bacterial richness are regarded to display a poorer overall health status and a more pronounced inflammatory phenotype (60). Our microbiome analysis revealed a significant disparity in the gut bacterial richness and composition in tinnitus, compared with HC. Among them, patients with tinnitus exhibited a lower alpha-diversity score, indicating a less diverse microbiota. Beta-diversity analysis also indicated substantial differences in the microbial composition between these two groups. Furthermore, we focused on the changes in specific taxa. For example, the Firmicutes/Bacteroidetes ratio, recognized as intestinal dysbiosis (61, 62), is increased in patients with tinnitus, indicating the imbalance of gut microbiota in tinnitus.

Manipulating gut microbiota through probiotics is associated with reduced inflammation in auditory conditions such as sensorineural hearing loss and otitis media (63, 64). Given that tinnitus falls within the category of auditory disorders, we found the gut microbiota profile in patients with tinnitus exhibits a pro-inflammatory pattern. Certain beneficial gut probiotics, such as Lactobacillaceae (48, 49) and *Akkermansia* (65, 66), were found to be enriched in healthy individuals, whereas some potentially pathogenic bacteria, including *Aeromonas* and *Acinetobacter* (46, 47), were significantly more prevalent in the tinnitus cohort. In particular, *Akkermansia* is a crucial probiotic with the potential to serve as a next-generation beneficial bacterium (67). Moreover, *Akkermansia* has the capability to produce a novel tripeptide Arg-Lys-His, which is capable of directly binding to Toll-like receptor 4 (TLR4) and inhibiting TLR4 signal transduction in immune cells (68). Therefore, *Akkermansia* exhibits promising effects in attenuating the progression of various pathological conditions associated with disruptions in immune system regulation and metabolic imbalances (e.g., type 2 diabetes, obesity, hepatic steatosis, cancer therapies) (50, 67). Although there is currently no mechanistic study elucidating the role of *Akkermansia* in tinnitus, our research suggests the possibility that a decreased abundance of this bacteria may contribute to heightened neuroinflammation in patients with tinnitus. We also observed a significant decrease in the relative abundance of *Prevotella* in patients with tinnitus. Alterations in *Prevotella* can influence the generation of pro-inflammatory cytokines (53), potentially contributing to neuroinflammation within the CNS (54). Furthermore, *Prevotella* has been reported to potentially correlate with a positive mood, and its relative abundance was higher in healthy volunteers (69, 70). However, the specific mechanism of how these gut microbiota regulate tinnitus needs further investigation.

Another hypothesis regarding the mechanism of tinnitus involves an imbalance between excitatory and inhibitory neurotransmitters, leading to increased self-firing in the auditory cortex (10–14). *Bifidobacterium adolescentis* and *Bifidobacterium dentium* have the ability to enhance the production of γ-aminobutyric acid (GABA), an inhibitory neurotransmitter (30, 31). Therefore, it is suggested that these two bacteria may play a role in the development of tinnitus (63). However, our study did not observe a significant difference at the species level between individuals with tinnitus and HCs. This discrepancy may be attributed to limitations in microbiological detection techniques, which cannot accurately differentiate between species. Additionally, the limited scale of our study and other bacteria with potential for producing neurotransmitters may have contributed to this result.

Substrate co-metabolism and metabolic exchange are the main pathways through which the gut microbiota interacts in a human host (23, 71). An imbalance in the gut microbiota may influence the transformation of dietary substrates, consequently leading to disturbances in serum metabolic profiles (72). Our findings suggest that serum metabolites play a role in the pathogenesis and pathophysiology of tinnitus. First, our PLS-DA has confirmed the presence of serum metabolic disturbances in the tinnitus group. A total of 89 tinnitus-associated serum metabolites were further identified, with the majority falling under the categories of lipids, lipid-like molecules, and organoheterocyclic compounds. There was a significant correlation between tinnitus and dysregulation of lipid metabolism, including serum levels of total cholesterol, triglyceride, low-density lipoprotein, and high-density lipoprotein (73, 74). A large-scale investigation into specific metabolites revealed a positive correlation between tinnitus and several metabolites, including C38:6 phosphatidylethanolamine (PE), C52:6 triglyceride, C36:4 PE, and C40:6 PE.

In contrast, our study did not identify a significant disparity in the levels of C36:4 PE between individuals with tinnitus and those without, while other metabolites were undetectable. However, we observed a notable decrease in C34:2 PE levels among individuals with tinnitus. This discrepancy may be attributed to the relatively smaller sample size in our study and potential influences from varying dietary habits across different countries within the sampled populations.

Specific lipid species play a crucial role as primary structural components of cellular membranes (73), whereas non-structural lipid molecules are widely acknowledged for their essential functions in the brain as specific receptor ligands and precursors of bioactive metabolites (53). Phosphatidylethanolamines, being the second most abundant phospholipids in cells, play a pivotal role in protein production, oxidative phosphorylation, and membrane fusion (73). They also serve as precursors to other lipids, and their imbalance is associated with neurodegenerative diseases (75, 76). Moreover, tinnitus has been significantly correlated with phosphatidylethanolamines (73), and lower levels of plasma PE among individuals exposed to occupational noise who have experienced hearing loss (77). However, the relationship of lipid metabolites and tinnitus remains entirely unclear. Further lipidomic studies are necessary to explore the potential use of serum lipids as therapeutic targets.

We have also identified tinnitus-related metabolic functional pathways associated with the regulation of neuroinflammation, such as tryptophan metabolism (51, 52) and arachidonic acid metabolism (78, 79). The kynurenine pathway is the major route of tryptophan metabolism, involved in inflammation and excitatory neurotransmission; tryptophan can also be converted to 5-hydroxytryptamine in central neurons and enterochromaffin cells (51). Therefore, it is widely recognized that various bioactive compounds derived from tryptophan metabolism play regulatory roles in diverse physiological processes, including inflammation and neurological function (51, 52). Furthermore, disruption in tryptophan metabolism has been shown to lead to several nervous system diseases, including depression, schizophrenia, Alzheimer’s disease, and Parkinson’s disease (51, 52, 80, 81). Given these functions of tryptophan metabolism, it has the potential to participate in the inflammation of auditory neurons and imbalance of neurotransmitters, which may contribute to the self-firing of auditory neurons associated with persistent tinnitus.

Based on the correlation analysis between the gut microbiota and serum metabolites, we observed a significant positive correlation between tinnitus-enriched genera (e.g., *Rhodococcus*, *Aeromonas*, and *Phyllobacterium*) and elevated serum metabolites in the tinnitus group. Conversely, tinnitus-depleted probiotics (e.g., *Lactobacillus*, *Lactococcus*, and *Akkermansia*) showed a positive correlation with tinnitus-depleted serum metabolites, some of which have been reported to possess anti-inflammatory properties. We have also discovered two bacterial modules (B6 and B22) that are enriched in tinnitus, suggesting their potential crucial roles in the development of tinnitus. PICRUSt2 allowed us to predict the representative function of tinnitus-related gut microbiota. Specifically, L-tryptophan degradation pathways and the fatty acid salvage were significantly upregulated in the tinnitus group, consistent with our metabolic analysis.

Our comprehensive multi-omics investigation provides a thorough overview of disturbances in the gut microbiota and related serum metabolites in patients with tinnitus. Based on these findings, we introduce the novel concept of the “gut-brain-ear axis” for the first time. Dysregulation of the gut microbiota leads to alterations in serum metabolites, and prolonged exposure to such a state may induce changes in auditory-related brain regions. Abnormal excitability or inflammatory damage to neurons decreases the stability of the auditory pathway and increases the susceptibility to tinnitus. Once inducible factors, such as peripheral auditory pathway damage, result in acute tinnitus, the changes mentioned above disrupt the negative feedback mechanism, and involvement of the limbic circuitry allows abnormal signals toward chronicity and emotional association. These findings first illustrated the potential involvement of the gut microbiota in the pathogenesis of tinnitus. We also provided a fresh perspective for tinnitus treatment, which may target specific gut taxa and serum metabolites mentioned in our study, supported by further functional studies.

Despite the robustness of our findings, our study has certain limitations. The relatively small sample size may have obscured the changes in the gut microbiota and serum metabolic profiles in tinnitus. Larger studies are necessary to validate our conclusions, and further functional experiments should be conducted to confirm our hypothesis about the “gut-brain-ear axis.” Furthermore, as a cross-sectional study, we only assessed microbial and metabolomic features at baseline without monitoring dynamic changes. Some potential confounding factors, such as diet, lifestyle, and social status, could influence gut microbiota composition, thus introducing bias into the correlation analysis. Additionally, the direct association between the gut microbiota and tinnitus requires validation through animal experiments, and further analysis of brain function is necessary to uncover the pathomechanisms underlying chronic tinnitus.

## Data Availability

The raw data from 16S sequencing have been deposited in the NCBI Sequence Read Archive (SRA) database (http://www.ncbi.nlm.nih.gov/sra) with the accession number PRJNA1181518. All data needed to evaluate the conclusions in this paper are presented in the paper and the supplemental material. The data sets used and/or analyzed during the current study are available from the corresponding authors on reasonable request. The underlying code for this study is not publicly available but may be made available to qualified researchers on reasonable request from the corresponding authors.

## SUPPLEMENTAL MATERIAL

Table S1 spectrum.01878-24-s0001.docx The quality of sequencing method.

Table S2 spectrum.01878-24-s0002.docx Different gut taxa at genus level.

Table S3 spectrum.01878-24-s0003.docx Significant microbiome correlated with clinical traits.

Table S4 spectrum.01878-24-s0004.docx Significant serum metabolites correlated with clinical traits.

ASM does not own the copyrights to Supplemental Material that may be linked to, or accessed through, an article. The authors have granted ASM a non-exclusive, world-wide license to publish the Supplemental Material files. Please contact the corresponding author directly for reuse.
