# Supplementary material for: Characteristic alterations of gut microbiota and serum metabolites in patients with chronic tinnitus: a multi-omics analysis
Source: Microbiol Spectr. 2024 Nov 18;13(1):e01878-24. doi: 10.1128/spectrum.01878-24 (PMC11705945; doi:10.1128/spectrum.01878-24)
Supplement: Table S1 — The quality of sequencing method. [file spectrum.01878-24-s0001.docx]

**Supplemental table 1. The quality of sequencing method.**

Samples from healthy control were labeled from C_1 to C_30 and those form patients with tinnitus were labeled from T_1 to T_70. Raw_Tags mean raw sequence data. Valid_Tags mean available sequence data. Valid mean the percentage of available data. Q20% mean the percentage of samples whose quality reached Q20. Q30% mean the percentage of samples whose quality reached Q30. GC% mean the percentage of G/C bases in sequence data.

| Sample | Raw_Tags | Raw_Bases | Valid_Tags | Valid_Bases | Valid% | Q20% | Q30% | GC% |
| --- | --- | --- | --- | --- | --- | --- | --- | --- |
| C_1 | 56011 | 23.18M | 56011 | 23.18M | 100.00 | 98.06 | 94.60 | 51.72 |
| C_2 | 51267 | 21.29M | 51267 | 21.29M | 100.00 | 97.62 | 93.48 | 51.44 |
| C_3 | 54760 | 22.77M | 54760 | 22.77M | 100.00 | 97.96 | 94.25 | 50.87 |
| C_4 | 63243 | 26.50M | 63243 | 26.50M | 100.00 | 97.81 | 93.95 | 51.28 |
| C_5 | 44877 | 18.58M | 44877 | 18.58M | 100.00 | 97.68 | 93.87 | 52.30 |
| C_6 | 61223 | 25.71M | 61223 | 25.71M | 100.00 | 97.56 | 93.43 | 51.20 |
| C_7 | 61613 | 25.84M | 61613 | 25.84M | 100.00 | 97.93 | 94.34 | 51.40 |
| C_8 | 43769 | 18.32M | 43769 | 18.32M | 100.00 | 97.73 | 94.08 | 53.02 |
| C_9 | 61565 | 25.42M | 61565 | 25.42M | 100.00 | 98.24 | 94.94 | 51.80 |
| C_10 | 62335 | 26.04M | 62335 | 26.04M | 100.00 | 98.02 | 94.44 | 51.24 |
| C_11 | 52553 | 21.57M | 52553 | 21.57M | 100.00 | 98.13 | 94.85 | 53.49 |
| C_12 | 63043 | 26.27M | 63043 | 26.27M | 100.00 | 98.03 | 94.54 | 51.19 |
| C_13 | 50083 | 20.77M | 50083 | 20.77M | 100.00 | 97.90 | 94.26 | 51.44 |
| C_14 | 59067 | 24.45M | 59067 | 24.45M | 100.00 | 98.04 | 94.50 | 51.82 |
| C_15 | 52715 | 21.74M | 52715 | 21.74M | 100.00 | 97.83 | 94.12 | 52.10 |
| C_16 | 52770 | 21.71M | 52770 | 21.71M | 100.00 | 97.65 | 93.76 | 52.06 |
| C_17 | 64075 | 26.39M | 64075 | 26.39M | 100.00 | 98.09 | 94.62 | 52.51 |
| C_18 | 58242 | 23.91M | 58242 | 23.91M | 100.00 | 97.88 | 94.11 | 53.70 |
| C_19 | 65719 | 27.20M | 65719 | 27.20M | 100.00 | 98.04 | 94.46 | 50.63 |
| C_20 | 62722 | 25.82M | 62722 | 25.82M | 100.00 | 97.82 | 94.05 | 52.85 |
| C_21 | 72906 | 30.64M | 72906 | 30.64M | 100.00 | 97.63 | 93.72 | 50.92 |
| C_22 | 57507 | 23.56M | 57507 | 23.56M | 100.00 | 97.97 | 94.47 | 52.30 |
| C_23 | 40327 | 16.70M | 40327 | 16.70M | 100.00 | 97.43 | 93.25 | 50.33 |
| C_24 | 59422 | 25.00M | 59422 | 25.00M | 100.00 | 97.74 | 93.73 | 51.19 |
| C_25 | 61761 | 25.39M | 61761 | 25.39M | 100.00 | 98.02 | 94.28 | 53.19 |
| C_26 | 57465 | 23.75M | 57465 | 23.75M | 100.00 | 97.77 | 93.68 | 50.93 |
| C_27 | 40035 | 16.44M | 40035 | 16.44M | 100.00 | 97.59 | 93.60 | 51.70 |
| C_28 | 58218 | 23.79M | 58218 | 23.79M | 100.00 | 97.56 | 93.28 | 52.87 |
| C_29 | 52586 | 21.57M | 52586 | 21.57M | 100.00 | 97.89 | 94.25 | 51.91 |
| C_30 | 56717 | 23.30M | 56717 | 23.30M | 100.00 | 97.61 | 93.40 | 51.98 |
| T_1 | 55726 | 23.04M | 55726 | 23.04M | 100.00 | 97.88 | 94.02 | 53.09 |
| T_2 | 56225 | 23.40M | 56225 | 23.40M | 100.00 | 96.76 | 90.93 | 49.33 |
| T_3 | 49399 | 20.29M | 49399 | 20.29M | 100.00 | 97.85 | 94.05 | 51.22 |
| T_4 | 59754 | 24.63M | 59754 | 24.63M | 100.00 | 97.83 | 93.89 | 54.38 |
| T_5 | 55588 | 23.05M | 55588 | 23.05M | 100.00 | 97.61 | 93.31 | 50.44 |
| T_6 | 46325 | 19.17M | 46325 | 19.17M | 100.00 | 97.77 | 93.97 | 51.78 |
| T_7 | 63261 | 26.06M | 63261 | 26.06M | 100.00 | 97.97 | 94.30 | 52.33 |
| T_8 | 54475 | 22.74M | 54475 | 22.74M | 100.00 | 97.78 | 93.97 | 51.52 |
| T_9 | 45694 | 18.71M | 45694 | 18.71M | 100.00 | 97.66 | 93.66 | 51.59 |
| T_10 | 40751 | 16.89M | 40751 | 16.89M | 100.00 | 97.08 | 92.21 | 50.73 |
| T_11 | 62133 | 25.87M | 62133 | 25.87M | 100.00 | 97.86 | 94.09 | 51.81 |
| T_12 | 43274 | 17.69M | 43274 | 17.69M | 100.00 | 97.35 | 93.11 | 52.65 |
| T_13 | 53808 | 22.56M | 53808 | 22.56M | 100.00 | 97.51 | 92.86 | 52.22 |
| T_14 | 56753 | 23.57M | 56753 | 23.57M | 100.00 | 97.98 | 94.36 | 51.57 |
| T_15 | 55037 | 22.58M | 55037 | 22.58M | 100.00 | 97.86 | 93.99 | 53.74 |
| T_16 | 48645 | 20.11M | 48645 | 20.11M | 100.00 | 97.52 | 93.30 | 50.25 |
| T_17 | 58775 | 24.50M | 58775 | 24.50M | 100.00 | 98.26 | 95.08 | 51.51 |
| T_18 | 58507 | 24.36M | 58507 | 24.36M | 100.00 | 97.29 | 92.30 | 50.78 |
| T_19 | 58831 | 24.06M | 58831 | 24.06M | 100.00 | 97.94 | 94.35 | 52.80 |
| T_20 | 53157 | 21.67M | 53157 | 21.67M | 100.00 | 97.66 | 93.64 | 51.91 |
| T_21 | 64000 | 26.62M | 64000 | 26.62M | 100.00 | 97.47 | 92.70 | 51.34 |
| T_22 | 55457 | 22.87M | 55457 | 22.87M | 100.00 | 98.01 | 94.42 | 51.33 |
| T_23 | 54021 | 22.39M | 54021 | 22.39M | 100.00 | 97.92 | 94.19 | 51.44 |
| T_24 | 102412 | 42.89M | 102412 | 42.89M | 100.00 | 97.42 | 93.25 | 50.84 |
| T_25 | 57046 | 23.32M | 57046 | 23.32M | 100.00 | 97.96 | 94.22 | 52.08 |
| T_26 | 42704 | 17.67M | 42704 | 17.67M | 100.00 | 97.14 | 92.13 | 50.80 |
| T_27 | 57271 | 24.12M | 57271 | 24.12M | 100.00 | 97.93 | 94.34 | 50.75 |
| T_28 | 58000 | 24.50M | 58000 | 24.50M | 100.00 | 97.59 | 93.31 | 50.28 |
| T_29 | 48573 | 20.06M | 48573 | 20.06M | 100.00 | 97.46 | 92.82 | 50.70 |
| T_30 | 52582 | 22.11M | 52582 | 22.11M | 100.00 | 97.98 | 94.34 | 51.27 |
| T_31 | 45769 | 19.14M | 45769 | 19.14M | 100.00 | 97.72 | 93.71 | 49.37 |
| T_32 | 73579 | 30.95M | 73579 | 30.95M | 100.00 | 97.60 | 93.60 | 50.75 |
| T_33 | 54450 | 22.34M | 54450 | 22.34M | 100.00 | 97.69 | 93.38 | 52.09 |
| T_34 | 69627 | 29.20M | 69627 | 29.20M | 100.00 | 97.66 | 93.52 | 51.50 |
| T_35 | 59583 | 24.92M | 59583 | 24.92M | 100.00 | 97.99 | 94.39 | 50.57 |
| T_36 | 64396 | 26.13M | 64396 | 26.13M | 100.00 | 97.86 | 94.07 | 52.78 |
| T_37 | 49372 | 20.67M | 49372 | 20.67M | 100.00 | 96.48 | 90.55 | 51.94 |
| T_38 | 41649 | 17.20M | 41649 | 17.20M | 100.00 | 97.57 | 93.64 | 52.92 |
| T_39 | 58089 | 24.61M | 58089 | 24.61M | 100.00 | 97.80 | 93.87 | 53.81 |
| T_40 | 43461 | 18.18M | 43461 | 18.18M | 100.00 | 97.27 | 92.84 | 50.68 |
| T_41 | 51788 | 21.70M | 51788 | 21.70M | 100.00 | 97.61 | 93.47 | 51.01 |
| T_42 | 54834 | 22.50M | 54834 | 22.50M | 100.00 | 97.34 | 92.49 | 51.18 |
| T_43 | 60207 | 25.15M | 60207 | 25.15M | 100.00 | 97.82 | 94.03 | 51.51 |
| T_44 | 57751 | 23.95M | 57751 | 23.95M | 100.00 | 97.69 | 93.63 | 53.62 |
| T_45 | 60015 | 24.70M | 60015 | 24.70M | 100.00 | 97.23 | 92.28 | 51.83 |
| T_46 | 44804 | 18.52M | 44804 | 18.52M | 100.00 | 97.82 | 94.15 | 51.02 |
| T_47 | 56488 | 23.00M | 56488 | 23.00M | 100.00 | 98.03 | 94.46 | 51.78 |
| T_48 | 59942 | 24.60M | 59942 | 24.60M | 100.00 | 97.91 | 94.25 | 52.19 |
| T_49 | 65115 | 27.15M | 65115 | 27.15M | 100.00 | 98.11 | 94.58 | 52.31 |
| T_50 | 65510 | 27.20M | 65510 | 27.20M | 100.00 | 97.03 | 91.74 | 52.49 |
| T_51 | 51075 | 21.09M | 51075 | 21.09M | 100.00 | 97.92 | 94.32 | 51.73 |
| T_52 | 62498 | 25.72M | 62498 | 25.72M | 100.00 | 98.04 | 94.64 | 54.80 |
| T_53 | 58307 | 24.28M | 58307 | 24.28M | 100.00 | 97.52 | 92.92 | 52.12 |
| T_54 | 51015 | 21.27M | 51015 | 21.27M | 100.00 | 97.94 | 94.26 | 50.71 |
| T_55 | 61207 | 25.03M | 61207 | 25.03M | 100.00 | 97.88 | 94.04 | 50.66 |
| T_56 | 64581 | 26.72M | 64581 | 26.72M | 100.00 | 97.91 | 94.17 | 50.86 |
| T_57 | 60367 | 24.91M | 60367 | 24.91M | 100.00 | 97.74 | 93.57 | 53.34 |
| T_58 | 55029 | 22.57M | 55029 | 22.57M | 100.00 | 97.26 | 92.16 | 53.31 |
| T_59 | 56953 | 23.40M | 56953 | 23.40M | 100.00 | 97.92 | 94.17 | 52.17 |
| T_60 | 60567 | 24.73M | 60567 | 24.73M | 100.00 | 97.82 | 93.99 | 52.28 |
| T_61 | 63234 | 25.93M | 63234 | 25.93M | 100.00 | 97.95 | 94.40 | 53.07 |
| T_62 | 52280 | 21.43M | 52280 | 21.43M | 100.00 | 97.65 | 93.88 | 52.62 |
| T_63 | 55038 | 22.61M | 55038 | 22.61M | 100.00 | 98.01 | 94.48 | 51.86 |
| T_64 | 42431 | 17.73M | 42431 | 17.73M | 100.00 | 97.35 | 93.10 | 50.25 |
| T_65 | 64324 | 27.12M | 64324 | 27.12M | 100.00 | 97.97 | 94.43 | 52.31 |
| T_66 | 58729 | 24.12M | 58729 | 24.12M | 100.00 | 97.97 | 94.45 | 52.29 |
| T_67 | 58324 | 23.87M | 58324 | 23.87M | 100.00 | 97.93 | 94.34 | 52.31 |
| T_68 | 61201 | 25.53M | 61201 | 25.53M | 100.00 | 97.61 | 93.60 | 51.57 |
| T_69 | 45426 | 18.73M | 45426 | 18.73M | 100.00 | 97.62 | 93.84 | 52.18 |
| T_70 | 55720 | 22.95M | 55720 | 22.95M | 100.00 | 97.84 | 94.15 | 52.14 |
