# Supplementary material for: Characteristic alterations of gut microbiota and serum metabolites in patients with chronic tinnitus: a multi-omics analysis
Source: Microbiol Spectr. 2024 Nov 18;13(1):e01878-24. doi: 10.1128/spectrum.01878-24 (PMC11705945; doi:10.1128/spectrum.01878-24)
Supplement: Table S2 — Different gut taxa at genus level. [file spectrum.01878-24-s0002.docx]

**Supplemental table 2. Different gut taxa at genus level.**

Different gut taxa were identified between patients with tinnitus and healthy control with a threshold of q-value < 0.05 and |log2FC| > 2. Wilcoxon test. The mean abundance of taxa was displayed in these two groups, respectively. If this taxa exhibited enrichment in tinnitus group, it would be labeled as ‘up’.

| Genus | log2FC | wilcox.pvalue | qvalue | Significance | Regulation | Mean_C | Mean_T |
| --- | --- | --- | --- | --- | --- | --- | --- |
| g__Rhodococcus | -7.07 | 0.0000 | 0.0000 | yes | up | 0.0005 | 0.0672 |
| g__Pseudomonas | -4.58 | 0.0000 | 0.0000 | yes | up | 0.0003 | 0.0072 |
| g__Vibrio | -3.87 | 0.0000 | 0.0000 | yes | up | 0.0059 | 0.0860 |
| g__Sphingomonas | -3.71 | 0.0000 | 0.0000 | yes | up | 0.0010 | 0.0131 |
| g__Aeromonas | -3.70 | 0.0000 | 0.0000 | yes | up | 0.0015 | 0.0195 |
| g__Pseudoalteromonas | -3.38 | 0.0000 | 0.0000 | yes | up | 0.0077 | 0.0802 |
| g__Ralstonia | -3.33 | 0.0000 | 0.0000 | yes | up | 0.0014 | 0.0141 |
| g__Lactiplantibacillus | -2.94 | 0.0000 | 0.0000 | yes | up | 0.0016 | 0.0123 |
| g__Brevundimonas | -2.85 | 0.0000 | 0.0000 | yes | up | 0.0124 | 0.0891 |
| g__Alloprevotella | -2.53 | 0.0000 | 0.0000 | yes | up | 0.0769 | 0.4436 |
| g__Phyllobacterium | -Inf | 0.0000 | 0.0000 | yes | up | 0.0000 | 0.0082 |
| g__Testudinibacter | -4.16 | 0.0001 | 0.0012 | yes | up | 0.0006 | 0.0107 |
| g__Acinetobacter | -3.42 | 0.0002 | 0.0021 | yes | up | 0.0011 | 0.0118 |
| g__Marinomonas | -Inf | 0.0004 | 0.0038 | yes | up | 0.0000 | 0.0035 |
| g__Shewanella | -5.21 | 0.0009 | 0.0073 | yes | up | 0.0001 | 0.0037 |
| g__Betaproteobacteria_unclassified | -Inf | 0.0047 | 0.0325 | yes | up | 0.0000 | 0.0112 |
| g__Ochrobactrum | -Inf | 0.0047 | 0.0325 | yes | up | 0.0000 | 0.0025 |
| g__Methylobacterium-Methylorubrum | -Inf | 0.0047 | 0.0325 | yes | up | 0.0000 | 0.0024 |
| g__Paramuribaculum | 2.10 | 0.0000 | 0.0000 | yes | down | 0.0279 | 0.0065 |
| g__Quinella | 2.10 | 0.0000 | 0.0000 | yes | down | 0.0047 | 0.0011 |
| g__Anaerotignum | 2.15 | 0.0000 | 0.0000 | yes | down | 0.0124 | 0.0028 |
| g__Eisenbergiella | 2.16 | 0.0000 | 0.0000 | yes | down | 0.1806 | 0.0405 |
| g__Leptotrichia | 2.17 | 0.0000 | 0.0000 | yes | down | 0.0193 | 0.0043 |
| g__MBNT15_unclassified | 2.23 | 0.0000 | 0.0000 | yes | down | 0.0580 | 0.0124 |
| g__Coriobacteriaceae_UCG-002 | 2.24 | 0.0000 | 0.0000 | yes | down | 0.0123 | 0.0026 |
| g__Akkermansia | 2.28 | 0.0000 | 0.0000 | yes | down | 1.2871 | 0.2641 |
| g__Lachnospiraceae_NK4A136_grodown | 2.36 | 0.0000 | 0.0000 | yes | down | 0.4058 | 0.0793 |
| g__Neisseria | 2.40 | 0.0000 | 0.0000 | yes | down | 0.1625 | 0.0308 |
| g__Prevotellaceae_NK3B31_grodown | 2.41 | 0.0000 | 0.0000 | yes | down | 0.1772 | 0.0334 |
| g__Helicobacter | 2.41 | 0.0000 | 0.0000 | yes | down | 0.0712 | 0.0134 |
| g__Rikenellaceae_RC9_gut_grodown | 2.41 | 0.0000 | 0.0000 | yes | down | 0.0483 | 0.0091 |
| g__Anaerolineaceae_unclassified | 2.41 | 0.0000 | 0.0000 | yes | down | 0.0117 | 0.0022 |
| g__Selenomonas | 2.54 | 0.0000 | 0.0000 | yes | down | 0.0070 | 0.0012 |
| g__Murimonas | 2.69 | 0.0000 | 0.0000 | yes | down | 0.0084 | 0.0013 |
| g__Muribaculum | 2.71 | 0.0000 | 0.0000 | yes | down | 0.0551 | 0.0084 |
| g__Candidatus_Saccharimonas | 2.73 | 0.0000 | 0.0000 | yes | down | 0.0279 | 0.0042 |
| g__Dubosiella | 2.77 | 0.0000 | 0.0000 | yes | down | 0.0538 | 0.0079 |
| g__HT002 | 2.95 | 0.0000 | 0.0000 | yes | down | 0.0898 | 0.0116 |
| g__Acetatifactor | 2.95 | 0.0000 | 0.0000 | yes | down | 0.0674 | 0.0087 |
| g__Ligilactobacillus | 2.96 | 0.0000 | 0.0000 | yes | down | 0.4878 | 0.0628 |
| g__Lactobacillus | 2.99 | 0.0000 | 0.0000 | yes | down | 0.8504 | 0.1067 |
| g__Lactococcus | 3.08 | 0.0000 | 0.0000 | yes | down | 0.0254 | 0.0030 |
| g__Prevotellaceae_UCG-001 | 3.23 | 0.0000 | 0.0000 | yes | down | 0.0103 | 0.0011 |
| g__Enterorhabdus | 3.24 | 0.0000 | 0.0000 | yes | down | 0.0284 | 0.0030 |
| g__Prevotellaceae_UCG-003 | 3.57 | 0.0000 | 0.0000 | yes | down | 0.0095 | 0.0008 |
| g__Oribacterium | 4.03 | 0.0000 | 0.0000 | yes | down | 0.0049 | 0.0003 |
| g__Aminicenantales_unclassified | 4.39 | 0.0000 | 0.0000 | yes | down | 0.0021 | 0.0001 |
| g__Pyramidobacter | 7.07 | 0.0000 | 0.0000 | yes | down | 0.4428 | 0.0033 |
| g__Chloroflexi_unclassified | 3.39 | 0.0001 | 0.0012 | yes | down | 0.0021 | 0.0002 |
| g__Aggregatibacter | 3.45 | 0.0001 | 0.0012 | yes | down | 0.0730 | 0.0067 |
| g__Filifactor | Inf | 0.0001 | 0.0012 | yes | down | 0.0012 | 0.0000 |
| g__Prevotellaceae_Ga6A1_grodown | 2.53 | 0.0002 | 0.0021 | yes | down | 0.0052 | 0.0009 |
| g__Capnocytophaga | 2.81 | 0.0003 | 0.0030 | yes | down | 0.0021 | 0.0003 |
| g__Moraxella | 2.73 | 0.0004 | 0.0038 | yes | down | 0.0053 | 0.0008 |
| g__Treponema | Inf | 0.0005 | 0.0045 | yes | down | 0.0015 | 0.0000 |
| g__Parvibacter | Inf | 0.0005 | 0.0045 | yes | down | 0.0011 | 0.0000 |
| g__Pedosphaeraceae_unclassified | Inf | 0.0005 | 0.0045 | yes | down | 0.0009 | 0.0000 |
| g__Rikenella | 2.04 | 0.0007 | 0.0060 | yes | down | 0.0037 | 0.0009 |
| g__Arthrobacter | 2.39 | 0.0009 | 0.0073 | yes | down | 0.0021 | 0.0004 |
| g__Absconditabacteriales_(SR1)_unclassified | 2.22 | 0.0015 | 0.0118 | yes | down | 0.0014 | 0.0003 |
| g__Gaiellales_unclassified | Inf | 0.0019 | 0.0146 | yes | down | 0.0006 | 0.0000 |
| g__Campylobacter | 2.42 | 0.0022 | 0.0167 | yes | down | 0.0363 | 0.0068 |
| g__Oscillospira | 2.22 | 0.0042 | 0.0305 | yes | down | 0.0587 | 0.0126 |
| g__4-29-1_unclassified | 2.81 | 0.0042 | 0.0305 | yes | down | 0.0014 | 0.0002 |
| g__Bacteroidales_unclassified | 2.24 | 0.0061 | 0.0405 | yes | down | 0.0289 | 0.0061 |
| g__Geobacter | Inf | 0.0075 | 0.0467 | yes | down | 0.0010 | 0.0000 |
| g__Desulfuromonadales_unclassified | Inf | 0.0075 | 0.0467 | yes | down | 0.0009 | 0.0000 |
| g__Rokubacteriales_unclassified | Inf | 0.0075 | 0.0467 | yes | down | 0.0006 | 0.0000 |
| g__TRA3-20_unclassified | Inf | 0.0075 | 0.0467 | yes | down | 0.0004 | 0.0000 |
| g__Sideroxydans | Inf | 0.0075 | 0.0467 | yes | down | 0.0004 | 0.0000 |
