# Supplementary material for: Characteristic alterations of gut microbiota and serum metabolites in patients with chronic tinnitus: a multi-omics analysis
Source: Microbiol Spectr. 2024 Nov 18;13(1):e01878-24. doi: 10.1128/spectrum.01878-24 (PMC11705945; doi:10.1128/spectrum.01878-24)
Supplement: Table S3 — Significant microbiome correlated with clinical traits. [file spectrum.01878-24-s0003.docx]

**Supplemental table 3. Significant microbiome correlated with clinical traits.**

Gut taxa, which significantly related with clinical traits, were identified using Spearman test. Correlation coefficient and P value were both displayed.

| Microbiome Name | Clinical Traits | Correlation | P_value |
| --- | --- | --- | --- |
| g__Lactobacillus | Sleep | -0.259614852 | 0.029980708 |
| g__Lachnospiraceae_NK4A136_group | Sleep | -0.411027063 | 0.000408451 |
| g__Eisenbergiella | Sleep | -0.26242138 | 0.028187943 |
| g__Mucispirillum | Sleep | -0.301873809 | 0.011093765 |
| g__Rhodococcus | Anxiety | 0.264568469 | 0.026878172 |
| g__Rhodococcus | Sleep | 0.287709106 | 0.015730511 |
| g__Tuzzerella | Sleep | -0.331679701 | 0.005032616 |
| g__Tuzzerella | Duration | 0.252841885 | 0.034705173 |
| g__HT002 | Sleep | -0.311552874 | 0.008654817 |
| g__Acetatifactor | Sleep | -0.408937937 | 0.000440018 |
| g__MBNT15_unclassified | Sleep | -0.336331433 | 0.004417228 |
| g__Muribaculum | Sleep | -0.277430094 | 0.020061399 |
| g__Dubosiella | Sleep | -0.245795094 | 0.040264823 |
| g__Rikenellaceae_RC9_gut_group | Sleep | -0.388522927 | 0.00088834 |
| g__Weissella | THI | -0.250729008 | 0.036300467 |
| g__Paramuribaculum | Sleep | -0.295340017 | 0.013059619 |
| g__Candidatus_Saccharimonas | Sleep | -0.243902759 | 0.041877608 |
| g__Enterorhabdus | Loudness | -0.262236762 | 0.028303033 |
| g__Enterorhabdus | Sleep | -0.305740154 | 0.010055939 |
| g__Lactococcus | Loudness | -0.295068003 | 0.013147611 |
| g__Phyllobacterium | Anxiety | 0.251502666 | 0.035709381 |
| g__Phyllobacterium | Sleep | 0.291456412 | 0.014365377 |
| g__Anaerotignum | Sleep | -0.300853041 | 0.01138282 |
| g__Coriobacteriaceae_UCG-002 | Sleep | -0.274675427 | 0.021381789 |
| g__Coriobacteriaceae_UCG-002 | Duration | 0.243241839 | 0.042453336 |
| g__RBG-13-54-9_unclassified | Sleep | -0.24742187 | 0.038919853 |
| g__Thermodesulfovibrionia_unclassified | Sleep | -0.28556354 | 0.016561265 |
| g__Murimonas | Sleep | -0.300998853 | 0.011341134 |
| g__Prevotellaceae_UCG-003 | Sleep | -0.250627938 | 0.036378285 |
| g__Subgroup_7_unclassified | Sleep | -0.304338502 | 0.010421971 |
| g__Prevotellaceae_Ga6A1_group | Sleep | -0.301144877 | 0.01129952 |
| g__Latescibacterota_unclassified | Sleep | -0.324482414 | 0.006134411 |
| g__Eubacterium]_nodatum_group | Sleep | -0.359144024 | 0.00226358 |
| g__Arthrobacter | Loudness | -0.245797563 | 0.040262754 |
| g__Arthrobacter | Sleep | -0.25073808 | 0.036293489 |
| g__Subgroup_18_unclassified | Sleep | -0.300940503 | 0.011357799 |
| g__Ochrobactrum | Sleep | 0.238246694 | 0.047019597 |
| g__Bacteroidetes_vadinHA17_unclassified | Sleep | -0.246017911 | 0.040078359 |
| g__Lachnospiraceae_UCG-006 | Sleep | -0.27652972 | 0.020485102 |
| g__Dialister | Loudness | -0.320192796 | 0.006887616 |
| g__Geobacteraceae_unclassified | Frequency | -0.33318691 | 0.004825403 |
| g__Parvimonas | Frequency | -0.300080943 | 0.011605778 |
| g__Candidatus_Solibacter | Frequency | -0.33318691 | 0.004825403 |
| g__Cellulosilyticum | Frequency | -0.33318691 | 0.004825403 |
| g__Beduinibacterium | Frequency | -0.33318691 | 0.004825403 |
