# Supplementary material for: Characteristic alterations of gut microbiota and serum metabolites in patients with chronic tinnitus: a multi-omics analysis
Source: Microbiol Spectr. 2024 Nov 18;13(1):e01878-24. doi: 10.1128/spectrum.01878-24 (PMC11705945; doi:10.1128/spectrum.01878-24)
Supplement: Table S4 — Significant serum metabolites correlated with clinical traits. [file spectrum.01878-24-s0004.docx]

**Supplemental table 4. Significant microbiome correlated with clinical traits.**

Serum Metabolites, which significantly related with clinical traits, were identified using Spearman test. Correlation coefficient and P value were both displayed.

| Metabolite Name | Clinical Trait | Correlation | P_value |
| --- | --- | --- | --- |
| .epsilon.-Caprolactam | Anxiety | 0.238862144 | 0.046436054 |
| Taurodeoxycholic acid | Anxiety | 0.32249717 | 0.006473473 |
| 1,1'-[1,12-Dodecanediylbis(oxy)]bisbenzene | Duration | 0.281795382 | 0.018111594 |
| Acylcarnitine 11:0 | Duration | -0.291024758 | 0.014517233 |
| .epsilon.-Caprolactam | Frequency | -0.242013733 | 0.043540522 |
| D-Sorbitol | Frequency | 0.23773883 | 0.047505662 |
| L-Iditol | Frequency | 0.340336516 | 0.003941809 |
| Norfloxacin | Frequency | -0.321791864 | 0.006597842 |
| Timolol | Frequency | -0.356984556 | 0.002416544 |
| 1,3-Dimethyluric acid | Loudness | 0.293521397 | 0.013657716 |
| 20a-Dihydroprogesterone | Loudness | 0.340986098 | 0.00386914 |
| 2-Hydroxy-5-methoxybenzoic acid | Loudness | -0.241709682 | 0.043813206 |
| L-Homoarginine | Loudness | 0.245900047 | 0.040176904 |
| N-(Phenylacetyl)-L-phenylalanine | Loudness | -0.235678688 | 0.049519917 |
| O-Benzyl-L-serine | Loudness | 0.279148835 | 0.019273321 |
| Phenylacetyl-L-glutamine | Loudness | 0.260546745 | 0.029375092 |
| .epsilon.-Caprolactam | Sleep | 0.314850124 | 0.007938444 |
| 2-Deoxyglucose | Sleep | -0.23853456 | 0.046745908 |
| PE 34:2; PE(16:0/18:2) | Sleep | -0.250737303 | 0.036294087 |
| Plasmenyl-PE 36:2; PE(P-18:0/18:2) | Sleep | -0.248606665 | 0.037963961 |
| Plasmenyl-PE 38:5; PE(P-18:0/20:5) | Sleep | 0.237566089 | 0.047671927 |
| Plasmenyl-PE 38:6; PE(P-16:0/22:6) | Sleep | -0.30167891 | 0.011148458 |
| 2-octenoylglycine | THI | -0.250470471 | 0.036499802 |
